# Long‐term outcomes of perianal fistulizing Crohn's disease in the biologic era

**DOI:** 10.1002/jgh3.12475

**Published:** 2020-12-20

**Authors:** Tanya Lee, Michael A Kamm, Sally Bell, Mark Lust, Steve Brown, Ola Niewiadomski, Chamara Basnayake, Emily Wright, Basil D'Souza, Rodney Woods, Shu Chen Wei, William Connell, Alexander Thompson, Eric Yong, Nik Sheng Ding

**Affiliations:** ^1^ Department of Gastroenterology St Vincent's Hospital Melbourne Victoria Australia; ^2^ Department of Medicine University of Melbourne Melbourne Victoria Australia; ^3^ Department of Colorectal Surgery St Vincent's Hospital Melbourne Victoria Australia; ^4^ Department of Internal Medicine National Taiwan University Hospital and College of Medicine Taipei Taiwan; ^5^ Department of Radiology St Vincent's Hospital Melbourne Victoria Australia

**Keywords:** biological therapy, Crohn's disease, magnetic resonance imaging, perianal fistula, tumor necrosis factor‐alpha

## Abstract

**Background and Aim:**

While the advent of biologic therapy has led to improved outcomes in perianal fistulizing Crohn's disease (pfCD), loss of response is common. Previous studies suggest that patients who achieve radiological healing (with healing of underlying tracts on magnetic resonance imaging [MRI]) have a longer duration of response. The aim of this study was to characterize MRI outcomes of pfCD at a specialist inflammatory bowel disease (IBD) unit and compare the long‐term clinical outcomes between patients achieving MRI and clinical healing.

**Methods:**

A retrospective analysis of perianal fistulizing Crohn's patients treated at one specialist IBD unit was performed. Records were reviewed for patient demographics, disease history, clinical assessments, investigation results, and disease flares. Clinical remission was defined as closure of all baseline fistula openings. Radiological healing was defined as the absence of any T2‐hyperintense sinuses, tracts, or collections. The primary end‐point was rate of MRI healing. The secondary outcome was defined as flare‐free period (time between clinical or radiological healing and patients' first signs/symptoms requiring therapy escalation).

**Results:**

A total of 93 patients were included, with a median follow‐up of 4.8 years (interquartile range, 2.4–6 years). Of 44 patients, 22 (50%) achieved clinical remission, while 15 of 93 (16%) achieved radiological healing. Of 22 patients, 10 (45%) with clinical remission had a subsequent disease flare (median time of 7 months) compared with 3 of 15 (20%) patients with MRI healing (median time of 3.6 years). Radiological healing was associated with a significantly longer flare‐free period (*P* = 0.01).

**Conclusion:**

Radiological healing occurs less commonly but represents a deeper form of healing, associated with improved long‐term clinical outcomes.

## Graphical abstract

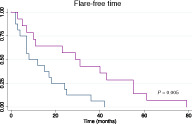

Patients with MRI healing have a significantly longer flare‐free period compared to those with only clinical healing (*P* = 0.005). This suggests that MRI healing is associated with improved clinical course compared to clinical remission, highlighting a potentially more sensitive and meaningful treatment target.

## Introduction

Perianal fistulizing Crohn's disease (pfCD) remains a significant clinical challenge due to its prevalence,^1^ debilitating symptoms, and effect on patient quality of life,^2^ as well as relative treatment resistance.^3^ Treatment is optimized with combined medical and surgical therapy.^4^ Since the introduction of anti‐tumor necrosis factor alpha (TNF) therapy for pfCD, patient outcomes appear to have improved, leading to fewer hospitalizations and invasive surgical procedures.^5^

Two broad categories of outcomes have been used to assess the efficacy of anti‐TNF therapy. In Sands *et al*.'s trial, clinical response was detected in 195 of 282 (69%) patients following infliximab induction.^6^ However, loss of response to maintenance therapy is common, occurring in 42% at a median of approximately 40 weeks.^7^

In contrast, radiologic assessment with magnetic resonance imaging (MRI) appears to be a more accurate and objective measure of disease activity, given the potential for persisting underlying fistula tracts on imaging, despite clinical healing.^8^, ^9^ However, few studies have characterized the radiological outcomes of pfCD, following biological therapy, with significant heterogeneity in patient cohorts in these studies.^9^ Furthermore, no universally accepted grading system exists to evaluate the severity of disease radiologically, with conflicting findings regarding the clinical utility of the Van Assche score, a composite radiological score that incorporates both inflammatory and anatomical features on MRI pelvis.^10^ While some studies have previously noted a significant difference in Van Assche score between clinical responders and nonresponders,^10^, ^11^, ^12^ others have since detected a significant decrease in score following treatment but not between clinical responders and nonresponders.^13^ In addition, some have demonstrated no significant difference in either pre‐ and posttreatment groups, nor between clinical responders and nonresponders, further highlighting the inconsistencies in current literature regarding MRI findings in this patient population.^14^

The long‐term outcomes of patients achieving MRI healing of pfCD have been reported in a limited number of studies.^15^, ^16^, ^17^ Tozer *et al*. concluded that achieving radiological healing did not signify that biological therapy should be ceased given that all patients who continued treatment maintained MRI remission compared to only 43% of those who stopped.^15^ While this suggests that MRI healing leads to improved clinical outcomes in the presence of maintenance therapy, it remains to be confirmed.

The aim of our study was twofold—to characterize the radiological outcome of pfCD patients and to compare the outcomes of patients achieving MRI healing to those achieving clinical remission, with the hypothesis that those achieving MRI healing will have a longer duration of response to treatment.

## Methods

### *Study design*

This was a retrospective cohort study of consecutive pfCD patients treated at one specialist inflammatory bowel disease (IBD) unit.

### *Participants*

**Figure 1:**
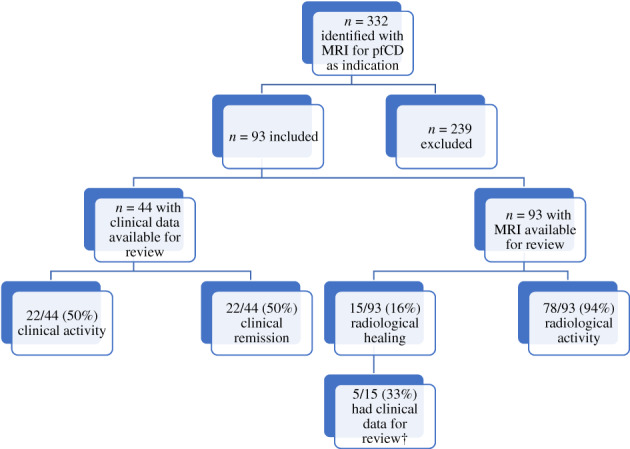
Inclusion and exclusion criteria for our study population. ^†^All patients with radiological healing, who had sufficient clinical data for review, also had clinical healing. MRI, magnetic resonance imaging; pfCD, perianal fistulizing Crohn's disease.

Patients were included in the study if they had an MRI diagnosis of a perianal fistula with a documented history of CD as established by clinical, radiological, histological, and/or endoscopic criteria. At least one follow‐up MRI needed to be available for review. Exclusion criteria included patients with an alternative cause for their perianal fistula, who did not have consultation notes from follow‐up appointments or investigation results available for review, and/or who had documented lack of compliance with therapy or frequent nonattendance to outpatient appointments (Fig. 1).

### *Data collection*

Potential patients were screened by obtaining the hospital Unit Record (UR) numbers of all patients who had received an MRI pelvis at St Vincent's Hospital, with the indication being pfCD. Patient records were accessed via the electronic medical records system using hospital UR numbers and were assessed for eligibility.

Medical records of patients who met the inclusion criteria were reviewed for the following information: demographics (patient age, gender, smoking status, body mass index), disease history (Montreal classification, fistula duration), any medical or surgical treatment(s) (current and past), disease flares (defined as any signs or symptoms requiring escalation in medical or surgical therapy), results of clinical assessments [number of external openings, Perianal Disease Activity Index (PDAI) scores], and investigation results (biochemistry, serum drug levels of biologic agents for therapeutic drug monitoring).

In addition, all MRI pelvis scans were reviewed by an experienced radiologist blinded to the clinical outcomes of the patients. Images were assessed for fistula number, location according to Parks classification; degree of T2‐hyperintensity; and presence or absence of supralevator extension, collections (≥3 mm), and rectal wall thickening. Using these parameters, the total Van Assche score was calculated. Scans taken prior to the commencement of a biologic agent were considered pretreatment images, while the latest images taken prior to the cessation of the biologic agent were considered posttreatment images.

### *Definition of outcomes*

Clinical closure of a fistula was defined as the absence of any discharge with gentle finger compression on examination. Clinical remission was defined as closure of all baseline fistulas, occurring at any time during the period of follow‐up. Radiological healing was defined as the absence of any T2‐hyperintense sinuses, tracts, or collections, corresponding to a Van Assche score of 0,^10^ in keeping with the current literature definition of MRI healing.^9^

The primary end‐point was the rate of radiological healing, on MRI, among pfCD patients treated with biologic therapy. The secondary outcome was defined as patients' “flare‐free period,” consisting of the amount of time between achieving remission (either clinical or radiological) and first flare (any signs or symptoms requiring escalation in treatment) or last patient follow‐up or cessation of biologic agent due to documented clinician loss of response.

### *Statistical methods*

The Stata software package (v.15.1.624; StataCorp, College Station, TX, USA) was used for all statistical analyses. Continuous values were summarized by medians and interquartile ranges (IQR) due to the skewed distribution of most variables. Numbers and percentages of patients in each category were used for categorical variables.

For univariate analysis, Mann–Whitney *U* tests were used to detect differences between continuous variables, while Fisher's Exact or Chi‐Squared test was used for categorical variables depending on expected sample sizes. The Wilcoxon Signed‐Rank test was used to compare paired samples.

Kaplan–Meier curves and log‐rank tests were used to compare the flare‐free periods between subgroups. A multivariate analysis was then carried out using a model of Cox regression with a continuously time‐varying parameter (being time to flare). A forward‐selection approach was employed, with clinically relevant parameters tested by log‐rank and entered into the model if *P* ≤ 0.2. A *P* < 0.05 was considered statistically significant.

### *Ethics*

Ethical approval was granted by the St Vincent's Hospital, Melbourne, Human Research Ethics Committee (Ethics ID LNR/18/SVHM/12) prior to project commencement.

## Results

### *Participants*

A total of 332 patients were initially identified as having had an MRI scan at St Vincent's Hospital, Melbourne, with pfCD being the primary indication. Of those, 173 were excluded due to a lack of adequate follow‐up and/or investigation results. Sixteen patients' diagnosis of Crohn's disease was equivocal; 48 patients were subsequently identified to have a perianal fistula secondary to another cause. Two further patients were excluded due to a lack of ongoing clinical data in the follow‐up period. Overall, 93 patients were included in the study.

### *Descriptive data*

Table 1 summarizes the basic demographics of the study population. There was a median follow‐up time of 4.8 years (IQR, 2.4–6 years). Of 93 patients, 5 (5%) had an initial documented CD diagnosis at St Vincent's Hospital, Melbourne, while others were referred from external sites. Of the 93 patients, 44 (48%) had results of clinical assessments for review. All patients had at least one follow‐up MRI; however, only 27 of 93 (29%) patients had a pretreatment scan available for analysis. Of 93 patients, 47 (51%) had received serum biologic drug level testing, with a total of 94 drug levels collected.

**Table 1:** Baseline demographics of patients included in our study

Characteristic	Number
Median age in years	38 (IQR, 21–63)
Females	53/93 (57%)
Median duration of fistula in years	13.5 (IQR, 9–20.5)
Age of diagnosis^†^	
<17 years	22/93 (24%)
15–40 years	63/93 (68%)
>40 years	7/93 (8%)
Location of disease^†^	
Terminal ileum	14/91 (15%)
Colonic	39/91 (43%)
Ileocolonic	37/91 (41%)
Upper disease only	0/91 (0%)
Perianal disease only	1 (1%)
Behavior of luminal disease^†^	
Nonstricturing, non‐penetrating	38/91 (42%)
Stricturing	41/91 (45%)
Penetrating	12/91 (13%)
Smoking status	
Smoker	19/93 (20%)
Ex‐smoker	25/93 (27%)
Non‐smoker	49/93 (53%)

† According to the Montreal Classification.

IQR, interquartile range.

**Figure 2:**
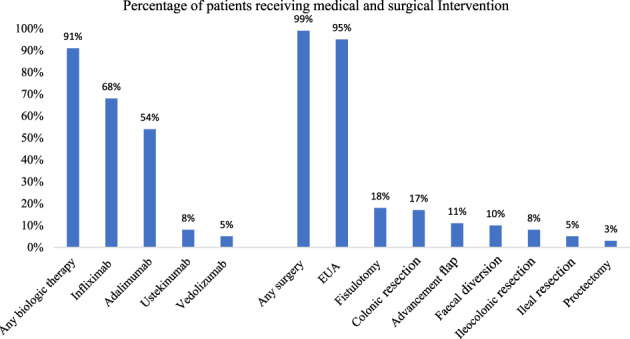
Summary of the medical and surgical interventions in our study population. Note that each patient may have had >1 biologic agent and therefore could be counted multiple times. Of 93 patients, 85 (91%) received biologic therapy, with infliximab being the most common agent. Of 93 patients, 92 (99%) had surgical intervention, the most common procedure being an Examination under Anesthesia (EUA).

Figure 2 summarizes the medical and surgical interventions for our cohort. Of 93 patients, 85 (91%) received biologic therapy. Infliximab was the most common agent, used in 63 of 93 (68%) patients during their treatment course. Of 93 patients, 92 (99%) had surgical intervention. The most common procedure was an examination under anesthesia (EUA), performed in 95% of the patients, with a median of 3 (IQR, 2–7) per patient.

### *Clinical outcomes*

Clinical remission was achieved in 22 of 44 (50%) of patients, 13 of 22 (60%) while on infliximab and 9 of 22 (40%) while on adalimumab. Of these, 10 of 22 (45%) had a flare in their disease following clinical remission, at a median of 7 months (IQR, 2.3–11 months). The remaining 12 (55%) did not meet the criteria for a flare since achieving healing for a median of 5.5 months (IQR, 0–17.3 months). There was no significant difference in age (*P* = 0.40), gender (*P* = 0.07), current smoking (*P* = 0.08), past smoking (*P* = 0.22), or location of luminal disease according to Montreal classification (*P* = 0.11) between patients who achieved clinical remission and those who did not.

There was a median baseline PDAI score of 8 (IQR, 6–9) and posttreatment score of 1 (IQR, 0–2), with a median duration of 3.5 years (IQR, 2–5.4 years) between pre‐ and posttreatment scores. A significantly lower PDAI score following treatment (*P* < 0.001) was detected.

### *Radiological outcomes*

Of 93 patients, 15 (16%) achieved radiological healing with a Van Assche score of 0. Table 2 summarizes the characteristics of patients who achieved MRI healing. Of 15 patients, 6 (40%) were on infliximab therapy, 6 (40%) were on adalimumab therapy, and 3 (20%) were not on biologic therapy. For those on biologic treatment, MRI healing was achieved at a median of 1.8 years (IQR, 1.2–4 years) following commencement. Of 15 patients, 12 (80%) did not meet the criteria for a flare after achieving healing for a median duration of 1.4 years (IQR, 0.5–3.6 years). Three of 15 (20%) patients did have a flare of their perianal disease at a median of 3.6 years (IQR, 3–4.3 years). Of the three patients who had a flare following MRI healing, two (67%) were not on maintenance biologic therapy, while one (33%) was on adalimumab maintenance therapy.

**Table 2:** Characteristics of patients who achieved radiological healing

Patient number	Age (years)	Gender	Biologic therapy	Relapse‐free time since achieving healing (months)
No flare since achieving MRI healing				
1	21	Female	Infliximab	33
2	26	Male	Adalimumab	5
3	30	Female	Infliximab	11
4	34	Male	Infliximab	56
5	35	Male	Adalimumab	7
6	39	Male	Infliximab	42
7	42	Female	Adalimumab	57
8	42	Female	Adalimumab	17
9	47	Female	Infliximab	5
10	60	Female	Infliximab	133
11	62	Female	—	29
12	63	Female	Adalimumab	2
Flare since achieving MRI healing				
13	25	Male	Adalimumab	61
14	41	Female	—	29
15	55	Male	—	43

Median pretreatment Van Assche score was 16 (IQR, 12–18). After a median duration of 3.2 years (IQR, 2–6 years), a posttreatment Van Assche score of 8 (IQR, 8–16) was obtained, which demonstrated a significant decrease (*P* = 0.008). In assessing the individual elements of the score, we found a significantly lower number of collections (*P* = 0.01) and degree of T2‐hyperintensity (*P* = 0.01) but no significant difference in the component of the score attributed to the number of tracts (*P* = 0.09). There was no significant difference in pretreatment Van Assche score (*P* = 0.81), posttreatment Van Assche score (*P* = 0.44), or magnitude of decrease in Van Assche score (*P* = 0.35) between those who achieved clinical remission and those who did not.

### *Comparison of clinical and radiological outcomes*

**Figure 3:**
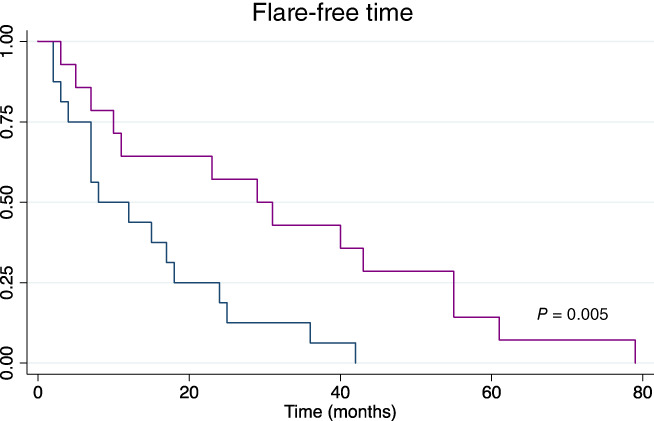
Kaplan–Meier curve comparing flare‐free period in patients with radiological healing with those achieving clinical remission. There was a significantly longer flare‐free period in patients with radiological healing on both univariate (*P* = 0.005) and multivariate (*P* = 0.01) analyses. This suggests magnetic resonance imaging healing is associated with improved clinical course compared to clinical remission. (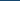), Clinical remission; (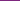), radiological healing.

The flare‐free period in patients with radiological healing was then compared to those who achieved only clinical healing, and a significantly longer duration was detected in patients with MRI healing on univariate analysis (*P* = 0.005). Figure 3 illustrates the flare‐free period in the two subgroups.

On univariate analysis, gender, biologic agent (infliximab or adalimumab), fistula duration, and smoking status had no significant impact on patients' flare‐free time, while patient age (*P* = 0.03) and type of healing (i.e. clinical *vs* radiological) were statistically significant. Patient age and healing type (clinical *vs* radiological) were then entered into multivariate analysis, with only healing type being statistically significant (*P* = 0.01).

### *Therapeutic drug monitoring*

Table 3 summarizes the serum biologic drug levels in our cohort. There was a median infliximab level of 3.3 μg/mL (IQR, 1.3–7.4 μg/mL) and a median adalimumab level of 3.2 μg/mL (IQR, 1.1–4.4 μg/mL) across 47 different patients and 94 separate drug levels. Only four of the patients who achieved radiological healing had biologic levels for review.

**Table 3:** Serum biologic drug levels in our study population

Radiological healing	Number of levels	Infliximab (median and IQR) in μg/mL	Adalimumab (median and IQR) in μg/mL
Healed	3 Infliximab 3 Adalimumab	4.95 (IQR, 4.13–5.78)	5.5 (IQR, 2.93–7.95)
Not healed	58 Infliximab 30 Adalimumab	3.3 (IQR, 1.3–8.2)	2.55 (IQR, 1.15–3.85)
Combined	61 Infliximab 33 Adalimumab	3.3 (IQR, 1.3–7.4)	3.2 (IQR, 1.1–4.4)

IQR, interquartile range.

## Discussion

Fistulizing Crohn's disease is a disabling phenotype, with wide‐ranging impacts on patient quality of life and limited treatment options. Current treatment regimens may result in clinical remission and, to a lesser degree, radiological healing in a smaller subset of patients. This study demonstrates that radiological healing is associated with a longer duration of response to biologics and may therefore represent a more clinically accurate and meaningful end‐point than clinical remission. A significant decrease in Van Assche score following treatment was also noted. Radiological follow‐up is an important component of patient care, and the findings of this study highlight the potential utility of individualized, MRI‐guided therapy in pfCD.

In this population, 22 of 44 (50%) pfCD patients had clinical remission, while 15 of 93 (16%) had radiological healing. Although we note that only 5 of 93 (5%) were diagnosed with Crohn's disease at our institution, with the remainder of patients being referred from external sites, our figures are consistent with one other publication that reported on rates of clinical remission and radiological healing, with similarly extensive follow‐up time, albeit with fewer patients.^13^ Of their 26 pfCD patients treated with infliximab, with median follow‐up of 4.9 years, 11 (42%) patients achieved clinical remission, while 2 of 14 (14%) had radiological healing, with the findings consistent with our own.

There was a significant difference in Van Assche score pre‐ and posttreatment but not between clinical responders and nonresponders. Notably, the components of the score that decreased significantly with treatment were the number of collections and degree of T2‐hyperintensity, while the number of tracts did not. This is in keeping with previous studies that reported on changes in the components of the Van Assche score, including the study by Van Assche *et al*.,^10^ which found that a decrease in T2‐hyperintensity was the most pronounced change in the score. In comparison, Savoye‐Collet *et al*. attributed the diminution of their population's Van Assche scores to improvement in the cavities component.^11^ Overall, our findings are consistent with those in the literature, in that biologic therapy is effective at diminishing signs of inflammation on MRI pelvis, as evidenced by resolution of collections and decreased T2‐hyperitensity; changes to the anatomical component of the Van Assche score (i.e. healing of tracts) was less common.

This is the first study to directly compare the outcome of patients who achieve deep radiological healing to those with clinical healing on examination. The majority of patients who achieved MRI healing remained flare‐free, while in 3 of the 15 (20%) patients, a subsequent flare despite radiological healing occurred at a median time of 3.6 years (IQR, 3–4.3 years). Our findings were consistent with those of Tozer *et al*., involving 41 pfCD patients and median follow‐up of 2.5 years, which reported that five of seven patients who remained on biologic therapy, following radiological healing, maintained response for the duration of the follow‐up.^15^ Our study demonstrated a significantly longer flare‐free period for the 15 patients who achieved MRI healing when compared to the 22 patients who achieved clinical healing on both univariate and multivariate analyses. Our findings suggest that MRI healing appears to be a less common, but more significant, end‐point than the widely employed outcome of clinical healing due to its association with a longer flare‐free period and duration of response to biological therapy. Furthermore, while clinical closure is a more readily assessible end‐point, its definition—the absence of draining from a fistula external opening with finger compression—is significantly more subjective and error prone.^8^ This indicates the importance of follow‐up MRIs in clinical practice, raising the utility of an MRI‐guided model of care using radiological healing as a therapeutic target. Further prospective trials would be required to confirm if treatment based on radiological findings leads to improved clinical outcomes.

This study detected relatively low serum biologic drug levels in our patients, with a median infliximab level of 3.3 μg/mL (IQR, 1.3–7.4 μg/mL) and a median adalimumab level of 3.2 μg/mL (IQR, 1.1–4.4 μg/mL). Previous studies have found that higher biologic levels are required to successfully treat fistulizing phenotypes of Crohn's Disease (CD) compared to luminal disease.^18^, ^19^ Yarur *et al*., in particular, noted that infliximab levels ≥10 μg/mL were associated with significantly higher rates of clinical remission, suggesting that the drug levels seen in our patient population were too low.^19^ Ideally, a comparison between levels of biologics in patients who achieved radiological healing compared to those who did not would have been performed. However, given that only four patients who achieved deep healing had serum levels tested, the sample size in this subgroup was too small to draw any conclusive results.

Our analysis of 93 pfCD patients, with a median of 4.8 years (IQR, 2.4–6 years) years of follow‐up, is the most extensive study of MRI outcomes in pfCD, both in terms of sample size and duration. However, a limitation is its retrospective nature, with inconsistencies in the amount of documentation and data available. We have previously noted discrepancies in outcomes assessed with relation to pfCD among the current literature, particularly pertaining to what constitutes clinical “response.”^9^ Hence, we limited the clinical outcomes assessed in our study to the stricter end‐point of clinical “remission,” which is more consistently defined as closure of all baseline draining fistulas within the current literature, to avoid introduction of potential treatment bias from clinicians. As a result, we found that only 44 of 93 (47%) of our patients had complete results of clinical assessments available for comparison.

There were also inconsistencies in the number of MRIs available for review, with only 27 of 93 (29%) patients with baseline scans available for comparison. Furthermore, an MRI is more likely to be organized when a patient has signs or symptoms or the clinician is suspicious of an underlying pathology; therefore, we may be capturing patients during more severe periods of their disease course. Similarly, with regard to therapeutic drug monitoring, patients may have been more likely to have their biologic levels assessed in a reactive fashion when they have a loss of response. Hence, there may be a bias toward indirectly selecting for lower drug levels due to the retrospective nature of the study.

In conclusion, MRI healing represents a more objective, and clinically relevant, end‐point than clinical remission and is associated with a significantly longer flare‐free period. Individualized treatment based on MRI, as opposed to clinically guided therapy, may result in better long‐term outcomes. Further prospective studies, with early MRI pelvis scans and escalation of therapy based on the findings, are required to confirm the benefit of radiological treatment targets.
